# Identifying individuals with complex and long-term health-care needs using the Johns Hopkins Adjusted Clinical Groups System: A comparison of data from primary and specialist health care

**DOI:** 10.1177/14034948231166974

**Published:** 2023-04-23

**Authors:** Rannei Hosar, Aslak Steinsbekk

**Affiliations:** Department of Public Health and Nursing, Norwegian University of Science and Technology (NTNU), Norway

**Keywords:** Population health management, risk stratification, adjusted clinical groups, ACG, complex and long-term health-care needs

## Abstract

**Aims:**

This study aimed to present the Johns Hopkins Adjusted Clinical Groups (ACG) System risk stratification profile of a total adult population of somatic health-care users when using data from either general practitioners (GPs) or hospital services and to compare the number and characteristics of individuals identified as having complex and long-term health-care needs in each data source.

**Methods:**

This was a registry-based study that included all adult residents (*N*=168,285) in four municipalities in Central Norway who received somatic health care during 2013. Risk profiles were generated using the ACG System based on age, sex and diagnoses registered by GPs or the local hospital. ACG output variables on number of chronic conditions, frailty and concurrent resource utilisation were chosen as indicators of complexity.

**Results:**

Nearly nine out of 10 (83.9%) of the population had been in contact with a GP, and 35.4% with the hospital. The mean number of diagnoses (3.0) was equal in both sources. A larger proportion of the population had higher risk scores in all variables except frailty when comparing hospital data to GP data. This was also found when comparing individuals identified as having complex and long-term health-care needs. A similar proportion of the population was found to have complex and long-term health-care needs (hospital 6.7%, GP 6.3%), but only one in five (21.5%) were identified in both data sets.

**Conclusions:**

**As data from GPs and hospitals identified mostly different individuals with complex and long-term health-care needs, combining data sources is likely to be the best option for identifying those most in need of special attention.**

## Introduction

Managing care for individuals with complex and long-term health-care needs [1] challenges the specialised, disease-oriented and reactive approach of traditional health-care systems [2]. Caring for such individuals includes treatment by a number of different health-care providers across medical specialties, professions and levels in the health-care system [3]. Consequently, they have an increased risk of experiencing loss of continuity and coordination, resulting in increased rates of adverse outcomes, institutionalisation and loss of function [4]. It has previously been suggested that interventions to improve integration of care can also improve the health outcomes of these individuals if correctly targeting and identifying high-risk individuals [5].

One approach for identification of these individuals is through risk stratification – a statistical process to detect characteristics associated with an increased chance of unwanted health outcomes [6]. It can be used to stratify the population according to their health status and identify specific subgroups, such as individuals with increased complexity in their health-care needs. The Johns Hopkins Adjusted Clinical Groups (ACG) System is a widely used risk stratification tool that utilises existing registry health data such as diagnoses and prescriptions [7,8]. The software has previously been validated for identifying individuals for care management interventions [9–12], and individuals with complex needs [1,9,13].

However, the source of input data can have an impact on the results of the risk stratification. Studies have investigated the performance of the ACG System using data from either a specialist or primary health-care setting independently [8,14] or used a combination of data from both sources [15,16]. Yet, we have not identified studies comparing the outcome of ACG risk stratification when using different data sources for the same population during the same period. This can be important, as local jurisdiction and health-care systems commonly do not allow for the combination of patient data beyond clinical care for individual patients. Thus, knowledge of the outcome of risk stratification when applying different data sources to identify individuals with long-term and complex health-care needs in the same population is needed.

The aims of this study were therefore to present the ACG risk stratification profile of a total adult population of somatic health-care users when using data from either general practitioners (GPs) or hospital services and to compare the number and characteristics of individuals identified as having complex and long-term health-care needs in each data source.

## Methods

### Study design

This was a registry-based study of all residents in four adjacent municipalities in Central Norway who had visited a GP or somatic hospital health-care service in 2013.

### Study setting

The study included one urban and three more rural municipalities. The urban municipality contains a university hospital that provides residents with general hospital health-care services.

Norway has a strong public health-care system that provides universal health care for all Norwegian residents. Each resident is provided a personal regular GP through a National Regular General Practitioner Scheme, and access to specialist health-care services is mainly referral based [17]. Specialist health-care services are mainly provided by hospitals with local or regional functions. The most specialised functions are located at university hospitals.

### Participants

All individuals aged ⩾18 years residing in the included municipalities registering one or more visit to a GP or somatic hospital health-care service between 1 January and 31 December 2013 were included in the study. Thus, individuals who had only been in contact with mental health care were not included, but data on both somatic and mental health-care visits were included for individuals who had visited both.

### The Johns Hopkins ACG System

The Johns Hopkins ACG System is a population case-mix system developed at Johns Hopkins University [7]. The system generates individual-level scores in a range of different output variables that quantify morbidity and predict concurrent and future health-care utilisation. The minimum input data requirements are age, sex and all known medical diagnoses for a set period of time, most commonly one year [7].

The ACGs are the key components and terminal groups of the ACG System algorithm. To arrive at these, diagnoses are first categorised based on likelihood of persistence, expected need and cost of procedures, and associated referral to specialist services, hospitalisation, disability and decreased life expectancy. The ACG System then clusters these categories by similar severity, likelihood of persistence and types of health-care services needed. Finally, each individual is assigned to one of 98 mutually exclusive ACGs according to their combination of diagnoses and demographic characteristics (age and sex). The system is described in further detail elsewhere [7,8].

### Data collection and processing

ACG System analyses were performed using registry data on age, sex and diagnosis codes registered in 2013. Diagnoses by GPs were registered as ICPC-2 codes, and diagnoses at the hospital were registered as ICD-10. These diagnosis codes were separated into two different data sets according to the data source and were analysed separately in the ACG System. Thus, the same individual could be present in both data sets.

Diagnoses from GPs were collected from the national Norwegian registry for reimbursement claims. The GP claims reimbursement for each consultation, and each claim must contain a minimum of one diagnosis code. Diagnoses from hospital health-care services were collected directly from the university hospital that also functions as the local hospital for all municipalities included in the study. This included diagnoses from all types of contact.

The data were anonymised and linked by first replacing the national ID number of each resident with a project ID number before linking them based on the project ID.

### Ethics approval and consent to participate

The study was approved by the Regional Committee for Medical and Health Research Ethics in Central Norway (2011/2047). As the study utilises secondary health-care data, active consent to participate was waived by the Regional Committee for Medical and Health Research Ethics.

### Identifying individuals with complex and long-term health-care needs

Five ACG variables were used to identify individuals with complex and long-term health-care needs (Table I). Three of the variables concern risk of high concurrent resource utilisation, and two of them concern relevant clinical conditions. An individual was categorised as having complex and long-term health-care needs if receiving a value over the cut-off limit in at least one variable.

**Table I.** Description of selected ACG variables retrieved from the Adjusted Clinical Groups System Version 11.0 Installation and Usage Guide [23] and cut-off values chosen in this study.

Variable	Descriptions	Cut-off value
RUB	Aggregations of ACGs based upon estimates of concurrent resource that are used to provide a way of separating the population into broad co-morbidity groupings as follows: 0 – No or only invalid diagnoses 1 – Healthy users 2 – Low 3 – Moderate 4 – High 5 – Very high	⩾4
Unscaled ACG concurrent risk	An estimate of concurrent resource use associated with a given ACG based on a reference database and expressed as a relative value. Each individual is assigned a weight based on their ACG-code.	⩾4
Unscaled concurrent risk	A concurrent total cost risk for this individual for the observation period. Based upon a regression model against a reference population (with a mean of 1.0), the predicted value is expressed as a relative weight.	⩾4
Frailty flag	A flag for any one of the diagnostic clusters that represent discrete conditions consistent with frailty (e.g. malnutrition, dementia, incontinence, difficulty in walking).	Yes
Chronic condition count	A count of EDCs containing trigger diagnoses indicating a chronic condition with significant expected duration and resource requirements.	⩾3

ACG: Adjusted Clinical Groups; RUB: resource utilisation band; EDC: expanded diagnosis cluster.

These five variables were chosen based on arguments from the current literature. Both frailty and multimorbidity have been described as characteristics of individuals with complex and long-term health-care needs [1]. The ACG System’s frailty flag has been found to identify an elderly population with the clinical characteristics of frailty correctly [18] and was applied directly. Disease counts is considered one of the most accurate measures for predicting health-care utilisation [19], and multimorbidity as a predictor in risk models has previously been found to increase accuracy [20]. Although there is no established consensus on how to define multimorbidity, a common definition is an individual with at least two or three chronic conditions. The present study therefore applied a cut-off value of a minimum of three in the chronic condition count of the ACG System.

The three remaining variables describing concurrent resource utilisation were chosen due to the documented association between increased resource consumption and complex health-care needs [21]. The resource utilisation bands (RUB; details in Table I) have previously been applied when identifying individuals with complex health-care needs [1], and the cut-off value of 4 is in accordance with previous research [1]. The cut-off values for unscaled concurrent risk (an individually calculated risk based on a regression model against a reference population) and unscaled ACG concurrent risk (the same risk applied to all individuals within the same ACG) were chosen to ensure including the population above the 95th percentile, which has previously been targeted as a population suited for case management [22].

All individuals registering a pregnancy delivery in the past year were assigned a RUB of 4 or higher in the ACG System [7]. As individuals with uncomplicated live births without co-morbidities were beyond our target group, these individuals were excluded from the analysis of those having complex and long-term health-care needs. This was done by excluding all individuals registered with ACG code 1711 [7].

### Statistical analyses

The ACG analyses were performed using version 11.0 of the Johns Hopkins ACG System Population Health Analytics software. Standard American weights were applied to increase international comparability. Stata v16.0 MP (StataCorp, College Station, TX) was used for further analysis. The characteristics of GP and hospital users were presented as descriptive statistics.

## Results

**Figure 1.**
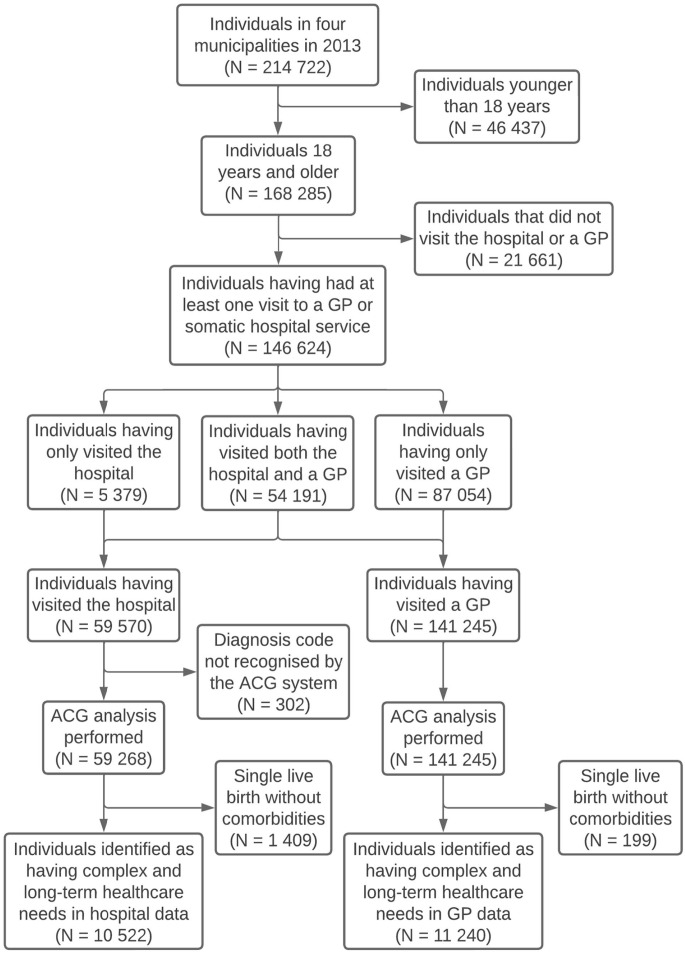
Flow chart of included participants.

Of the total adult population of 168,285, 146,624 (87.1%) registered a minimum of one visit to a GP or hospital (Figure 1). Of these, 59,570 (35.4%) had visited the hospital, and 141,245 (83.9%) had visited a GP.

### Risk stratification profile of the total population

The age and proportion of females were slightly higher among individuals who had visited the hospital than those who had visited a GP (Table II). Each individual was registered with an average of three unique diagnoses in both data sources. This means that the average number of diagnoses for each individual used as input in the ACG System was equal. However, the average number of chronic conditions was 0.4 times higher using hospital data.

**Table II.** Risk profile characteristics of the total population and of identified individuals complex and long-term health-care needs.

Characteristic	Total population		Identified as having complex and long-term health-care needs	
	Data source		Data source	
	Hospital (*N*=59,268)	General practitioner (*N*=141,245)	Hospital (*N*=11,240)	General practitioner (*N*=10,522)
Mean age (SD), years	50.9 (0.081)	47.0 (0.050)	60.1 (0.191)	61.0 (0.196)
Age range (years)				
18–39	20,206 (34.1%)	56,151 (39.7%)	2464 (21.9%)	2134 (20.3%)
40–59	17,472 (29.5%)	46,204 (32.7%)	2335 (20.8%)	2171 (20.6%)
60–79	16,614 (28.0%)	31,242 (22.1%)	4226 (37.6%)	4033 (38.3%)
80+	4976 (8.4%)	7648 (5.4%)	2215 (19.7%)	2184 (20.8%)
Sex				
Female	34,269 (57.9%)	77,885 (55.1%)	6767 (60.2%)	6612 (62.8%)
Male	24,937 (42.1%)	63,360 (44.9%)	4471 (39.8%)	3910 (37.2%)
Mean (SD) number of unique diagnoses	3.0 (0.012)	3.0 (0.006)	6.8 (0.039)	5.8 (0.028)
Mean number of unique chronic conditions	0.9 (0.005)	0.5 (0.002)	2.4 (0.017)	1.9 (0.013)
RUB				
RUB1	8051 (13.6%)	24,495 (17.3%)	43 (0.4%)	144 (1.4%)
RUB2	22,372 (37.8%)	50,358 (35.6%)	572 (5.1%)	532 (5.1%)
RUB3	22,244 (37.5%)	62,322 (44.1%)	5433 (48.3%)	5973 (56.8%)
RUB4	5587 (9.4%)	3907 (2.8%)	4178 (37.2%)	3708 (35.2%)
RUB5	1014 (1.7%)	163 (0.1%)	1014 (9.0%)	163 (1.5%)
Frailty flag				
Yes	1793 (3.0%)	3052 (2.2%)	1793 (16.0%)	3052 (29.0%)
No	57,475 (97.0%)	138,193 (97.8%)	9447 (84.0%)	7470 (71.0%)
Unscaled ACG concurrent risk				
0–0.49	33,275 (56.1%)	84,271 (59.7%)	911 (8.1%)	847 (8.1%)
0.50–0.99	13,954 (23.5%)	38,678 (27.4%)	3220 (28.6%)	2949 (28.0%)
1–1.99	5633 (9.5%)	14,442 (10.2%)	2112 (18.8%)	3069 (29.2%)
2–3.99	5360 (9.0%)	3653 (2.6%)	3951 (35.1%)	3454 (32.8%)
⩾4	1046 (1.8%)	201 (0.1%)	1046 (9.3%)	201 (1.9%)
Unscaled concurrent risk				
0–0.49	26,734 (45.1%)	100,229 (71.0%)	458 (4.1%)	1124 (10.7%)
0.5–0.99	8629 (14.6%)	19,523 (13.8%)	621 (5.5%)	1370 (13.0%)
1–1.99	8870 (15.0%)	12,438 (8.8%)	1232 (11.0%)	2968 (28.2%)
2–3.99	8738 (14.8%)	7092 (5.0%)	2660 (23.7%)	3097 (29.4%)
⩾4	6293 (10.6%)	1963 (1.4%)	6269 (55.8%)	1963 (18.7%)

Data shown as *n* (%), mean (SD) and difference in means for continuous variables and percentage point difference for proportions.

A larger proportion of the population presented with high scores in all variables, indicating complex and long-term health-care needs when comparing risk stratification based on diagnoses registered at the hospital to those registered by GPs. The only exception was a higher proportion of the population categorised as frail in data from GPs (difference in proportions 12.9%).

### Number of individuals identified as having complex and long-term health-care needs

A slightly higher number of individuals were identified as having complex and long-term health-care needs when applying data from the hospital, with 6.7% in hospital data and 6.3% in GP data (Table III). Looking at the specific variables, the highest number of individuals were identified by the multimorbidity variable in both data sources, followed by a high RUB. The mean number of ACG variables with a score above the cut-off limit was 0.4 times higher in hospital data than in GP data.

**Table III.** Number and proportion of individuals identified in each or both data sources.

Variable	Data source		
	Hospital	General practitioner	Number that received a high score in both data sources
Multimorbidity	5237	4174	1163
Frailty flag	1793	3052	515
High RUB	5192	3871	1125
High unscaled ACG concurrent risk	1046	201	37
High unscaled concurrent risk	6269	1963	1055
Summarised number of individuals	19,536	13,261	5621
Total number of unique individuals	11,240	10,522	3849
Proportion of unique individuals identified among all individuals present in the respective data source	19.0%	7.4%	–
Proportion of unique individuals identified in the adult population (*N*=168,285)	6.7%	6.3%	2.3%
Mean number (SD) of complexity criteria fulfilled	1.7 (0.010)	1.3 (0.006)	

**Figure 2.**
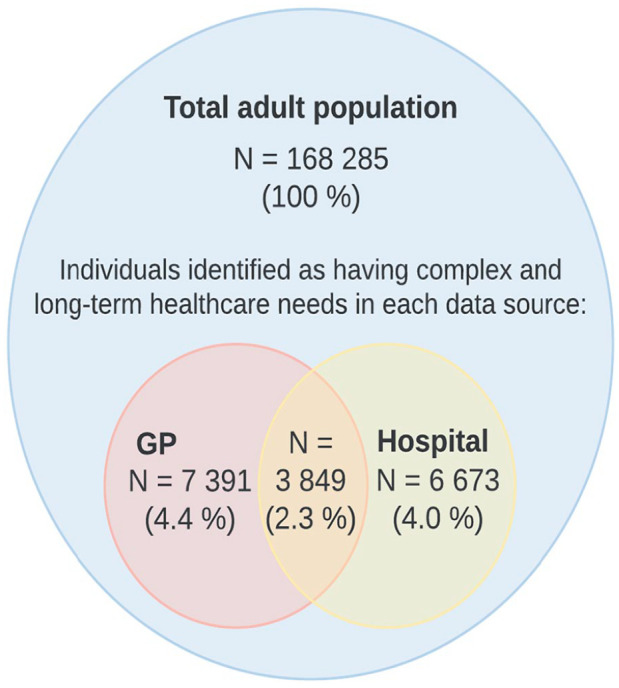
Proportion of total adult population identified as having complex and long-term health-care needs by source.

As shown in Figure 2 and Table III, using only hospital data identified 11,240 unique individuals, while using only data from GPs identified 10,522 as having complex and long-term health-care needs, given the chosen cut-off values. Among these identified individuals, 3849 (21.4%) were identified by both data sources.

### Characteristics of the individuals identified as having complex and long-term health-care needs

The age and sex distribution among those in the population identified as having complex and long-term health-care needs were similar in both sources (Table II). Individuals identified using hospital data generally had higher scores in the ACG variables than those identified using GP data. The exception was frailty, which was found in nearly twice the number of individuals in GP data.

The most frequently occurring diagnosis codes among the individuals identified as having complex and long-term health-care needs were similar in both data sets (Supplemental Material). Hypertension, atrial fibrillation/flutter, supervision of pregnancy, chronic obstructive pulmonary disease, diabetes mellitus, heart failure and urinary tract infection were all among the top 20 most frequent diagnoses in both sources.

## Discussion

Risk stratification scores based on data from hospital services yielded higher scores than data from GPs in all risk variables except frailty. Hospital registry data identified 6.3% of all individuals as having complex and long-term health-care needs compared to 6.7% when using GP data. However, there were substantial differences in who were identified when applying registry data from the two data sources, with only one fifth of the total number of identified individuals being identified in both sources.

### Validity of diagnosis codes

With the ACG System stratifying based on diagnosis codes registered for administrative purposes [7], the quality of the output is dependent on the validity of the registered diagnoses. Previous investigation of the validity of diagnostic codes in medical services claims in general have found the codes to have high specificity but to vary in sensitivity and to tend to underreport chronic conditions [23,24].

The quality of diagnosis codes registered in the Norwegian registry for reimbursement claims, which is applied in the present study as the source of GP data, has been found to correspond well with the content of patient records [25]. The Norwegian Patient Registry that routinely receives the same administrative data from Norwegian hospitals that was applied as the source of hospital data in the present study has been found to contain diagnosis codes of sufficient quality for epidemiological research [26]. In conclusion, both sources of data are assessed to hold a sufficient level of quality for risk stratification purposes.

However, the number of diagnosis codes available from each data source could potentially vary. One possible reason is that few reimbursement claims from GPs contain more than the one required diagnosis [25], while hospitals routinely report additional diagnoses as the basis for part of their economic reimbursement [27]. However, in the present study, the mean number of diagnoses for each individual registered in each data source was equal.

### Identification of individuals with complex and long-term health-care needs

No publications regarding which ACG variables are best suited to identify people with complex and long-term health-care needs were identified, nor which cut-off values to use. There are studies which have defined criteria for including individuals with complex needs in research studies. One example is Buja et al. [1,13] who applied the following inclusion criteria: individuals ⩾65 years of age, characterised as having complex health-care needs (without further specification) and with a RUB of ⩾4.

Although such studies give some indications, the question remains whether other variables could be better suited or whether the cut-off values are set too high or low. This needs further investigation, as applying appropriate indicators of complex and long-term health-care needs is a prerequisite for correctly targeting the individuals most likely to benefit from proactive interventions [5].

### Different data sources provide different results

Although the proportion of the population identified using each data source indicated that both sources are suitable, the groups identified by the two data sources included mostly different individuals. This is supported by findings that the prevalence of chronic conditions and multimorbidity varies across different data sources [24,28,29]. These studies have also found that the same individual can be registered with different chronic conditions in different data sources.

Given the finding that only one fifth were identified in both data sets, it seems reasonable to conclude both sources ought to be used in combination. Combining different sources has been suggested as a feasible alternative to overcome the problem of variation between data sources [24,28,29]. This option is appealing, as it seems to provide the most comprehensive foundation for correctly targeting the relevant group of individuals.

Nevertheless, it is not always possible to combine data sources. Although it is possible to gain access to linked data for research purposes, it is often not the case in clinical practice. Additionally, there are substantial differences between linking retrospective health registry data for research purposes and sharing patient journals in real time, as would be the preferred approach for identifying individuals with complex and long-term health-care needs in the clinical setting.

Combining sources also raises the question of how large a proportion of the population it is beneficial to identify. The traditional aim is 5% [30], and our study showed that each separate source identified roughly this desired proportion. It also showed that when adding up these individuals identified by each source, they constitute 10.6% of the total population. Still, they are identified using the same criteria and appear to be similar. This raises a discussion on which percent is the correct target level when using varying data sources and combinations of these, and if or how the data source needs to be accounted for.

Our study thus implies that more knowledge is needed about what proportion of the population should be targeted when identifying individuals with complex and long-term health-care needs. When doing so, the focus should be on identifying those for whom interventions are likely to have an impact [31] and who will benefit from interventions such as case management. Obtaining more information on this from both research and clinical practice will have implications for policy and practice.

### Strengths and limitations

The main strength of this study was the availability of data from two data sources covering the same population during the same period. Due to the completeness of the registries used and the limited private health-care sector [17,25,26], the sample represents all users of somatic health care in the population.

As discussed, the usage of secondary registry data makes the study vulnerable to inadequate coding of diagnoses. It is also possible that the one-year study period could exclude some diagnosis codes, and that providing additional detailed data on prescriptions and health-care utilisation would generate ACG output variables that would increase the accuracy of the identification of those individuals having complex and long-term health-care needs. Results might also have differed if other cut-off values for the included ACG variables had been applied. Although diagnosis codes concerning mental health care were included for all participants, one of the inclusion criteria was having made a minimum of one visit to a somatic health-care service. This would potentially exclude individuals with complex and long-term mental health-care needs if they did not receive somatic health care.

As described in the methods section, using the default weights of the ACG System increases international comparability. However, they are based on US costs. Although previous studies have found the ACG System to have a high adaptive capacity [32] and able to explain the cost of primary care in neighbouring country Sweden [33], we cannot exclude the possibility of differences in the relative cost of primary and specialist health care between the USA and Norway.

## Conclusions

Within the limitations of the study, using the same identification model with data from GPs and hospitals identifies similar proportions of the population as having complex and long-term health-care needs. However, as data from GPs and hospitals identified mostly different individuals, combining data sources is likely to be the best option for identifying those most in need of special attention.

## Supplemental Material

sj-docx-1-sjp-10.1177_14034948231166974 – Supplemental material for Identifying individuals with complex and long-term health-care needs using the Johns Hopkins Adjusted Clinical Groups System: A comparison of data from primary and specialist health care Supplemental material, sj-docx-1-sjp-10.1177_14034948231166974 for Identifying individuals with complex and long-term health-care needs using the Johns Hopkins Adjusted Clinical Groups System: A comparison of data from primary and specialist health care by Rannei Hosar and Aslak Steinsbekk in Scandinavian Journal of Public Health
