# Supplementary material for: Identifying individuals with complex and long-term health-care needs using the Johns Hopkins Adjusted Clinical Groups System: A comparison of data from primary and specialist health care
Source: Scand J Public Health. 2023 Apr 23;52(5):607–15. doi: 10.1177/14034948231166974 (PMC11292969; doi:10.1177/14034948231166974)
Supplement: sj-docx-1-sjp-10.1177_14034948231166974 – Supplemental material for Identifying individuals with complex and long-term health-care needs using the Johns Hopkins Adjusted Clinical Groups System: A comparison of data from primary and specialist health care [file sj-docx-1-sjp-10.1177_14034948231166974.docx]

**Supplementary material**

Supplementary table 1. Most frequent diagnoses (number of individuals with the diagnosis) among individuals identified as having complex and long-term healthcare needs in hospital and GP data respectively. Diagnoses are sorted according to frequency in each data source. Similar diagnoses from both hospital and GP data are marked in italic.

| **Hospital** | **General practitioner** |
| --- | --- |
| *Essential (primary) hypertension (1 686)* | *Hypertension uncomplicated (2 050)* |
| Single live birth (1 414) | No disease (1 869) |
| *Supervision of normal pregnancy, unspecified (1 365)* | *Cystitis/urinary infection other (1 298)* |
| *Supervision of other high-risk pregnancies (1 002)* | *Diabetes non-insulin dependent (1 290)* |
| Other specified orthopaedic follow-up care (879) | Incontinence urine (1 218) |
| Atherosclerotic heart disease (683) | *Atrial fibrillation/flutter (1 074)* |
| Other and unspecified symptoms and signs involving the nervous and musculoskeletal systems (677) | Disorder pregnancy/delivery, other (1 060) |
| Old myocardial infarction (659) | Health maintenance/prevention (944) |
| *Chronic obstructive pulmonary disease (unspecified) (623)* | Depressive disorder (921) |
| *Type 2 diabetes mellitus without complications (615)* | *Pregnancy (919)* |
| *Atrial fibrillation and atrial flutter, unspecified (607)* | General disease not otherwise specified (887) |
| Other physical therapy (578) | *Chronic obstructive pulmonary disease (751)* |
| *Heart failure, unspecified (577)* | Dementia (750) |
| *Urinary tract infection, site not specified (567)* | Sleep disturbance (692) |
| Chemotherapy session for neoplasm (552) | Upper respiratory infection acute (650) |
| Poisoning: Oxytocic drugs (545) | Hypothyroidism/myxoedema (619) |
| Angina pectoris, unspecified (453) | *Abdominal pain/cramps general (601)* |
| *Other and unspecified abdominal pain (450)* | Weakness/tiredness general (563) |
| Chest pain, unspecified (447) | *Heart failure (533)* |
| Bacterial pneumonia, unspecified (434) | Cough (532) |
